# Causal Deep Neural Network-Based Model for First-Line Hypertension Management

**DOI:** 10.1016/j.mcpdig.2023.10.001

**Published:** 2023-11-30

**Authors:** Lee Herzog, Ran Ilan Ber, Zehavi Horowitz-Kugler, Yardena Rabi, Ilan Brufman, Yehuda Edo Paz, Francisco Lopez-Jimenez

**Affiliations:** a K Health, New York, NY; b Department of Cardiovascular Medicine, Mayo Clinic, Rochester, MN

## Abstract

**Objective:**

To develop and validate a machine learning model that predicts the most successful antihypertensive treatment for an individual.

**Patients and Methods:**

The causal, deep neural network-based model was trained on data from 16,917 newly diagnosed hypertensive patients attending Mayo Clinic’s primary care practices from January 1, 2005, to December 31, 2021. Eligibility criteria included a diagnosis of primary hypertension, blood pressure and creatinine measurements before antihypertensive treatment, treatment within 9 months of diagnosis, and at least 1 year of follow up. The primary outcome was model performance in predicting the likelihood of a successful antihypertensive treatment 1 year from the start of treatment. Treatment success was defined as achieving blood pressure control with no moderate or severe adverse effects. Model validation and guideline agreement was assessed on 1000 patients.

**Results:**

In the training set of 16,917 participants (60.8±14.7 years; 8344 [49.3%] women), 33.8% achieved blood pressure control without moderate or severe adverse effects for at least a year with initial treatment. The most common treatment was angiotensin-converting enzyme inhibitor (39.1% average success), and the most successful was angiotensin-converting enzyme inhibitor-thiazide combination (44.4% average success). Our custom-built causal, deep neural network-based model exhibited the highest accuracy in predicting individualized treatment success with a precision of 51.7%, recall of 44.4%, and F1 score of 47.8%. Compared with actual physician practice on the validation set (77.9% agreement), the algorithm aligned with the Eighth Joint National Committee hypertension guidelines 95.7% of the time.

**Conclusion:**

A machine learning algorithm can accurately predict the likelihood of antihypertensive treatment success and help personalize hypertension management.

Hypertension is a major risk factor for cardiovascular disease, disability, and death globally. It is also one of the most common conditions managed in the primary care setting. Antihypertensive therapy has a well-established role in the reduction of cardiovascular morbidity and mortality. Clinical guidelines help guide the choice of antihypertensive drug therapy. The guidelines are typically derived from aggregated studies, with findings generalized to the level of the drug class. A meta-analysis of studies on the effect of hypertension treatments on different age groups found no difference in reduction of total major cardiovascular events by antihypertensive drug class.^1^ In addition, antihypertensive agents across different drug classes at equivalent dosages were shown to produce similar reductions in blood pressure.^2^ Consensus guidelines do suggest the use of specific drug classes for patients with certain comorbidities, for example, use of angiotensin-converting enzyme (ACE) inhibitors in patients with heart failure, previous myocardial infarction, diabetes, or proteinuric chronic kidney disease. Despite the presence of consensus guidelines and the similarity in long-term cardiovascular outcomes and blood pressure reduction with different drug regimens, there is wide variability in blood pressure response to treatment at the individual level.^3^ We have developed a causal, deep neural network (DNN)-based model for optimizing and individualizing treatment of hypertension in the primary care setting.

Deep learning, a subfield of machine learning, is a type of artificial intelligence that uses neural networks with multiple layers to automatically learn meaningful patterns from data.^4^ Recent use in the medical field includes recognition of hyperkalemia on electrocardiograms^5^ and creation of a differential diagnosis for common skin conditions.^6^ A causal framework is one that accounts for cause and effect between an intervention and its observed outcome. It has an advantage over a predictive framework that only accounts for associations. A predictive model for antihypertensive treatment, for instance, may learn that treatment with low dosages usually succeeds. If used incorrectly, such a model can lead to recommending low doses comprehensively to all patients. By contrast, a causal model will learn that the association between low dosages and success is because of the fact that low doses are given in mild hypertension cases. Such a model will not necessarily recommend low doses for all patients. Formally, causality is achieved by satisfying 3 conditions: positivity, exchangeability, and consistency.^7^ Our modeling framework fulfills these requirements.

To our knowledge, this is the first time a causal, DNN-based recommendation engine for hypertension management has been published or is being used in clinical practice. Our model optimization accounts for the success of achieving blood pressure control and the likelihood of adverse effects. Our algorithm results also deepen the level of treatment resolution beyond that of the clinical guidelines, providing personalized treatment recommendations at the drug class and specific drug and dosage levels.

## Methods

### Patients

The causal, DNN-based model was trained and validated using longitudinal data from patients attending Mayo Clinic’s primary care practices between 2005 and 2021. Inclusion criteria were a new diagnosis of primary hypertension by International Classification of Diseases (ICD)-10 code, established care in the primary care setting before the diagnosis of hypertension, treatment with an antihypertensive within 9 months of hypertension diagnosis, at least 1 year of follow-up post-treatment, blood pressure and creatinine measurements before initiation of antihypertensive treatment, and documented outcome measurements as defined below after treatment. Exclusion criteria included a new diagnosis of diabetes or prediabetes within a year of initiating antihypertensive treatment and prescription of a blood pressure treatment (ingredient and dosage pair) that existed less than twice in the data set.

### Model Variables and Data Extraction

#### Patient Variables

Patient characteristics extracted included demographic characteristic features (age, sex, and race), comorbid conditions (type II diabetes mellitus, chronic kidney disease, ischemic heart disease, tachyarrhythmia, congestive heart failure, gout, and asthma), and blood pressure and laboratory measurements. Conditions were defined on the basis of ICD-10 code. Tachyarrhythmia included ICD-10 codes for atrial fibrillation, atrial flutter, atrial tachycardia, unspecified tachycardia, and other cardiac arrhythmias such as ventricular fibrillation. The validity of using ICD-10 codes to capture diagnosis of conditions was assessed for a representative group of conditions (Supplemental Table 1, available online at https://www.mcpdigitalhealth.org/). 84% of the patients had at least 1 blood pressure reading, and 67% had at least 2 readings averaging ≥130 systolic or ≥80 diastolic within a year of primary hypertension diagnosis defining these patients as hypertensive per the American College of Cardiology 2017 guidelines.^8^ All patients in the data set were treated with an antihypertensive further confirming the hypertension diagnosis. To fulfill the mathematical criteria for a causal model^7^ all patients were required to have all features documented in the medical record. Cases for which the patient had no baseline blood pressure or creatinine measurements within a year of initial hypertension treatment were excluded. Data events during hospitalizations were excluded.

#### Blood Pressure Data Extraction

Ambulatory systolic and diastolic blood pressure measurements were extracted from the flowsheets table in the electronic health record (EHR). Measurements that were invasive, recorded during exercise, operation, anesthesia, or dialysis, taken in a position other than sitting, or on a body part other than the arm were excluded.

#### Medication Data Extraction

RxNorm was used as the medication identification code in the EHR. Blood pressure medications were mapped to antihypertensive drug classes on the basis of RxNorm codes using tables constructed by McDonough et al.^9^ Antihypertensive medications missing RxNorm codes in the EHR were manually mapped to RxNorm codes on the basis of medication name and dosage. All medications were then separated into their ingredient and dose components. Treatment equivalency could then be understood independently of whether the medication was given as separate pills or in combination. Medication frequency and prescription instructions from the EHR were used to determine daily treatment dosage. Order instructions were free text and the prescription instructions were extracted using natural language processing (eg, “take 2 pills 3 times a day”) to calculate the total treatment dosage. Medication start and stop dates were taken from prescription start and end dates, and a timeline was created for each ingredient-dosage unit.

### Outcomes

#### Blood Pressure Control

Treatment success was defined as achieving blood pressure control with no moderate or severe adverse effects for at least a year after primary antihypertensive treatment. Average blood pressure control was defined on the basis of guidelines from the Eighth Joint National Committee (JNC 8),^10^ the standard of care among primary care physicians at the time of model development. It was considered successfully controlled for patients <60 years if the average blood pressure was <140/90 mm Hg and never surpassed >160 mm Hg systolic or >90 mm Hg diastolic. For patients over 60 years old, successful blood pressure control was defined as an average <150/90 mm Hg and never surpassing >170 mm Hg systolic or >90 mm Hg diastolic. Blood pressure outcome measurements were included if they were collected 6-12 months from the start of treatment. If the average blood pressures during these time intervals were above JNC 8 blood pressure goals or a measurement passed our high threshold, the treatment was considered a failure. If the medication was discontinued before 1 year on the medication, the case was included as a failure of the treatment if blood pressures at least 2 weeks post-initial treatment and 6 months before the end of treatment were high compared with average and threshold levels. Cases with discontinued medications and controlled blood pressures before discontinuation were excluded as information about year-long success was missing. If the patient did not have a recorded blood pressure measurement within a year of initial treatment, for as long as the patient was maintained on the same prescription, the outcome blood pressures were calculated for up to 2 years and the earliest of the following intervals was used to determine blood pressure success: 12-14 months, 14-18 months, and 18-24 months. The earliest time frame with a calculated value was included as the blood pressure control outcome. At least 1 blood pressure reading was required to define treatment success. On average patients had 4 blood pressure measurements included in the blood pressure control outcome. Blood pressure readings during hospitalization were excluded.

### Adverse Effects

Failure of treatment because of adverse effects was defined at any point after initial treatment if the patient had hypotension (blood pressure <90 mm Hg systolic or <60 mm Hg diastolic), bradycardia (heart rate <50, with or without symptoms), serum creatinine increase (>30% relative to baseline), hypo or hypernatremia (<130 or >150 mEq/L), hypo or hyperkalemia (<3.6 or >5.1 mEq/L), elevated fasting glucose (>120 mg/dL), or moderate or severe adverse effects to the medication as documented in the medical record in the allergies table. Symptomatic adverse effects were categorized by level of adverse effect. Mild adverse effects such as diarrhea were not considered treatment failure, whereas moderate or severe symptoms such as cough for ACE inhibitor or edema with calcium channel blocker (CCB) were considered treatment failure. In the training set, 75% of cases with moderate and 85.1% of cases with severe adverse effects were linked to discontinuation of the medication within a month of reporting the adverse effect.

### Data Processing, Model Development, and Model Evaluation

Blood pressure, age, and creatinine were modeled as continuous variables. Sex, race, and presence of comorbid conditions were modeled as categorical variables. The treatment success rate was calculated at the ingredient and dosage levels. To present results at the class level, we aggregated results from the specific ingredients and weighed the success results according to the probability of each ingredient and dose being prescribed.

Three types of models were considered during model development. First, was a plain statistical model. Statistical models have the advantage that results are easily interpretable. However, results are limited when the number of features is large and there is an interest in recommendations at the individual level. This is because the relevant sample size for every individual tends to decrease exponentially with the number of features so the variance in success rate becomes very large. Second, we tested an XGBoost model. Lastly, we developed a custom DNN-based model. We experimented with a number of architectures and hyperparameter configurations. The parameters of the selected model are available in Supplemental Table 2, available online at https://www.mcpdigitalhealth.org/. A confidence score was also calculated for our model results.

In order to properly address causality, 3 assumptions have to be satisfied: positivity, exchangeability, and consistency. Positivity states that for every sub-population we can only compare between the treatments that are actually given to that sub-population. We account for this through our confidence threshold, which is designed to filter out positivity (or near positivity) violations. Exchangeability is when the potential outcome of an individual under a certain treatment does not depend on the actual treatment. This mathematical statement for dealing with confounders was satisfied by feeding the model with all features that may be correlated with the treatment. It is exchangeability that allows the comparison of different treatments in identical populations. Consistency means that there are no ambiguities in treatment definition. Our model operates at the ingredient and dosage level, even when results are presented at the ingredient or class levels, thereby fulfilling the requirements of consistency.

The model’s ability to accurately predict individualized antihypertensive treatment success was evaluated by comparing actual treatment success from blood pressure and adverse effect outcomes for each patient in the validation set to the model’s prediction of treatment success or failure given the patient and actual prescribed treatment. The model’s ability to infer the causal relationship between an antihypertensive treatment and blood pressure success was measured by calculating the agreement between the model’s top suggested drug class and the JNC 8 drug class recommendations for a given patient in the validation set. The JNC 8 guidelines recommend that individuals with chronic kidney disease be treated with ACE inhibitor or angiotensin receptor blockers (ARB), Black patients be treated with thiazide or CCB, and all others begin first-line treatment with an ACE inhibitor, ARB, thiazide, or CCB. The guidelines allow for the use of single or combination agents for first-line hypertension management.

### Statistical Analyses

Analysis for Table 1 was done using statistical functions from the python package tableone.^11^

**Table 1:** Baseline Patient Characteristics

Characteristic	Training Set	Validation Set	*P*
	(n=16,917)	(n=1000)	
Age (y), mean ± SD	60.8±14.7	60.7±14.1	.78
Age groups (y), n (%)			.56
18-39	1421 (8.4)	73 (7.3)	
40-49	2184 (12.9)	137 (13.7)	
50-59	4087 (24.2)	259 (25.9)	
60-69	4566 (27.0)	262 (26.2)	
70-79	2961 (17.5)	177 (17.7)	
80+	1698 (10.0)	92 (9.2)	
Female, n (%)	8344 (49.3)	519 (51.9)	.12
Ethnicity, n (%)			.91
White	15,641 (92.5)	927 (92.7)	
Black	551 (3.3)	33 (3.3)	
Other	725 (4.3)	40 (4.0)	
Systolic blood pressure pretreatment, mean ± SD, mm Hg	137.8±18.9	137.9±19.2	.89
Diastolic blood pressure pretreatment, mean ± SD, mm Hg	81.0±11.5	81.3±11.7	.55
Pretreatment blood pressure classification, n (%)			.52
Normal	3976 (23.5)	235 (23.5)	
Elevated	3897 (23.0)	229 (22.9)	
Hypertension stage 1	3412 (20.2)	185 (18.5)	
Hypertension stage 2	5632 (33.3)	351 (35.1)	
Comorbidities, n (%)			
Tachyarrhythmia	4972 (29.4)	281 (28.1)	.4
Type II diabetes	3647 (21.6)	210 (21.0)	.71
Ischemic heart disease	2593 (15.3)	151 (15.1)	.88
Asthma	1961 (11.6)	136 (13.6)	.06
Chronic kidney disease	1381 (8.2)	77 (7.7)	.65
Congestive heart failure	1126 (6.7)	64 (6.4)	.8
Gout	561 (3.3)	37 (3.7)	.57
None	7175 (42.4)	422 (42.2)	.92
Creatinine, mean ± SD, mg/dL	1.0±0.8	1.0±0.6	.06

### Data Availability

All model development and validation was conducted on deidentified data behind glass that was accessed with Mayo Clinic Platform_Discover.

## Results

**Figure 1:**
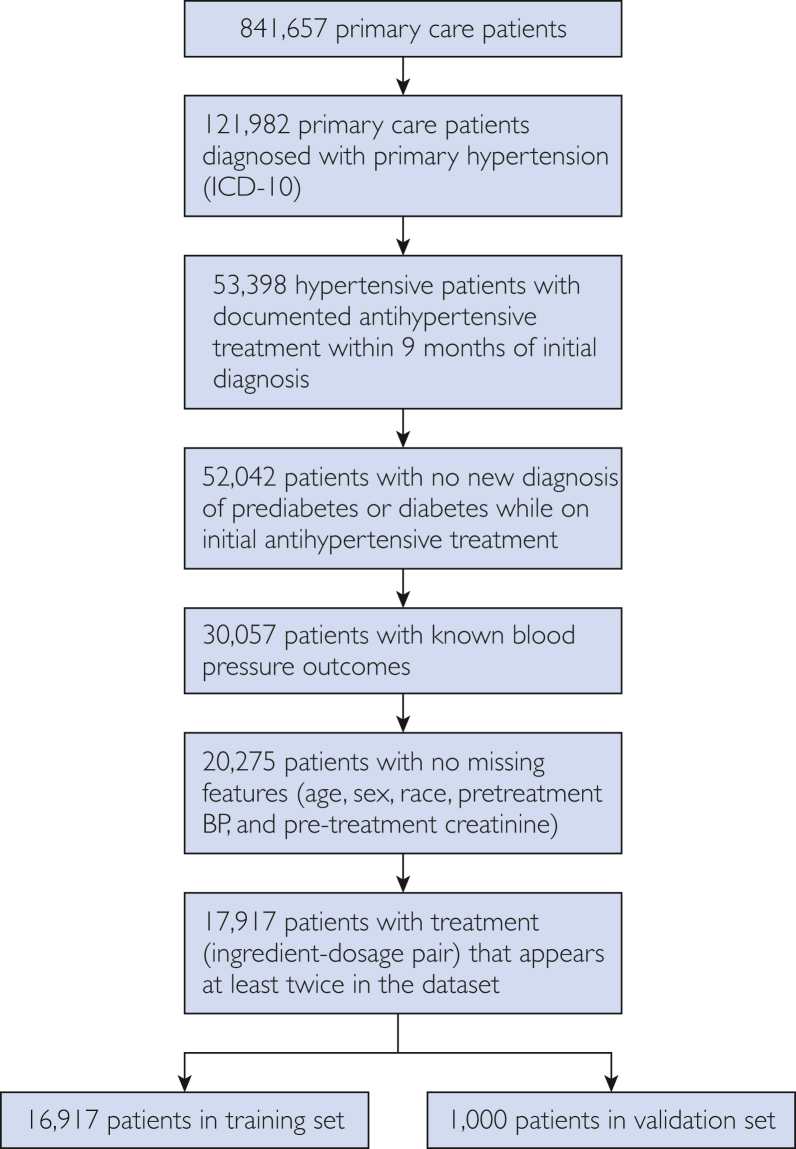
Data set creation. Schematic of the data set separation for training and testing of the model. No patients were allocated to more than one data set. BP, blood pressure.

A total of 121,982 primary care patients with primary hypertension were screened to identify the model development cohort. 17,917 patients fulfilled the inclusion criteria (Figure 1). A total of 16,917 patients were assigned to the model training set and 1000 patients to the validation set. The training and validation sets were mutually exclusive, with no patients appearing in more than 1 group. Table 1 shows the patient characteristics for the training and validation sets. The distributions of baseline factors in the 2 data sets were similar across features. The overall population had a mean age of 60.8 years, and 62.8% had at least 1 comorbidity. The most commonly used drug classes were ACE inhibitor (26.5%), β blocker (13.9%), thiazide (12.6%), CCB (11.3%), and ARB (8.7%). The average rate of success was 33.8%. Table 2 shows the average success rate per drug class in the training population. The ACE inhibitor-thiazide combination, ACE inhibitor monotherapy, and thiazide monotherapy were the most effective treatment classes. Supplemental Table 3, available online at https://www.mcpdigitalhealth.org/ shows the most likely treatment and success rate for that treatment on the basis of race and comorbidities in the training population.

**Table 2:** Treatment Likelihood and Success Rate by Drug Class in the Training Set

Drug Class	Treatment Likelihood (%)	Average Success Rate (%)
ACE inhibitor	26.5	39.1
β blocker	13.9	30.9
Thiazide	12.6	38.5
Calcium channel blocker	11.2	29.6
ARB	8.6	35.4
ACE inhibitor-thiazide combination	5.4	44.5

ACE, angiotensin-converting enzyme; ARB, angiotensin receptor blocker.

In the validation set, 78.0% of patients were treated with a single agent and 22.0% with 2 or more agents. It was observed that 34.2% of patients achieved blood pressure control for at least a year with no reported moderate or severe adverse effects with the first treatment alone. Supplemental Table 4, available online at https://www.mcpdigitalhealth.org/ compares the performance of different models on the validation set. Our causal, DNN-based model had the highest accuracy predicting individualized treatment success. The algorithm predicted patient-specific treatment success on the actual drug class prescribed in the validation set with a precision of 51.7%, recall of 44.4%, and F1 score of 47.8% (Supplemental Table 4, available online at https://www.mcpdigitalhealth.org/). The model was also able to distinguish between cases it was more and less confident in (Supplemental Figure 1, available online at https://www.mcpdigitalhealth.org/). When comparing the model’s performance on all validation cases to validation cases for which the model had medium or high confidence in the success prediction (418 cases), the performance on the validation set increased to precision 51.6%, recall 72.1%, and F1 60.1%. For cases for which the model had high confidence (173 cases), the performance increased to precision 58.1%, recall 91.9%, and F1 71.2%. When comparing the causal, DNN-based algorithm results to the JNC 8 guideline recommendations for the validation set cases, the percent agreement was 95.7% (Table 3).

**Table 3:** Percent Agreement with Guidelines on Validation Set

Method	Percent Agreement with Guidelines^a^ (%)
Physician practice^b^	77.9
XGBoost	85.0
Statistical model	88.5
Hypertension causal, deep neural network model	95.7

a Percentage of the validation cases for which there was agreement between the model’s top suggested drug class and the JNC 8 guideline recommended drug classes for the case given a patient’s race and comorbidities.

b Physician practice is defined as the actual treatment prescribed by the provider for a given validation case.

**Figure 2:**
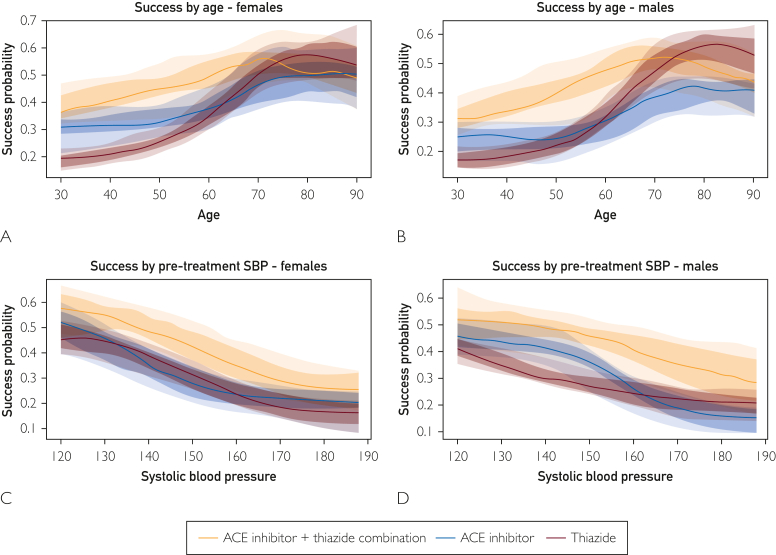
Treatment success likelihood for ACE inhibitor vs thiazide as monotherapy or combination therapy. Model prediction of success rates as a function of age (A and B) or systolic blood pressure (C and D) by treatment drug class (top 3 most successful drug classes per feature set). Representative cases shown in graphs are for a White individual with no comorbidities and serum creatinine level of 0.8 mg/dL (A, B, C, and D). Pretreatment blood pressure of 160/90 mm Hg (A and B). 50 year-old (C and D). The success prediction is composed of 20 models. Each was trained on 80% of the train set and was initiated with different weights. The median score of these models was considered to be the actual probability and is the dark line in the plots. The medium tones represent the area between the 25th and the 75th percentiles and the lighter tones represent the area between the 10th and 90th percentiles. SBP, systolic blood pressure.

The most successful antihypertensive treatment class identified by the model for White individuals was the ACE inhibitor-thiazide combination. The advantage of the combination over treatment with ACE inhibitors or thiazides as single agents was consistent across sexes (Figures 2A and B), pretreatment blood pressures (Figures 2C and D), and serum creatinine levels (Supplemental Figure 2, available online at https://www.mcpdigitalhealth.org/). Comparing the 2 most successful single agent drug classes ACE inhibitors and thiazides, the algorithm found that ACE inhibitors produced greater treatment success than thiazides for those less than 60 years old, whereas thiazides were more efficacious for older individuals. This treatment effect by age was consistent across sexes (Figures 2A and B).

The model suggested a consistent increase in success for Black individuals treated with thiazides relative to other single agents at all ages. The benefit of thiazides and CCBs over ACE inhibitors was most pronounced for Blacks over the age of 55 (Supplemental Figure 3, available online at https://www.mcpdigitalhealth.org/). As seen in Supplemental Figure 3, the confidence intervals for treatment success by drug class among Black individuals overlaps. A likely further separation of treatment success among classes is expected for a population with a larger sample of Black individuals.

## Discussion

The study findings suggest that a machine learning algorithm can accurately predict the most successful antihypertensive treatment for an individual. Our model results have high agreement with the JNC 8 hypertension clinical guidelines and move beyond the guideline recommendations, which allow for multiple possible classes of treatment. Our model suggests the patient-specific treatment class with the highest predicted likelihood of achieving blood pressure control without causing moderate or severe adverse effects. We can also discriminate between cases for which the model has high confidence in the success measure and those for which it is less certain. In addition, the model expands beyond the hypertension guidelines by providing results not only at the drug class resolution level but at the ingredient and dosage levels as well.

Our modeling framework fulfilled causality requirements and the data used to train the model was consistent with known clinical outcomes in hypertensive patients. The average success rate among patients receiving initial treatment for hypertension of 33.8% in our training population is supported by the literature.^12^^,^^13^ In the ALLHAT study, 90% of participants were on antihypertensives pre-randomization but only 27% had controlled blood pressures at baseline. At the year 1 follow-up 55% had blood pressures <140/90 mm Hg.^13^ In addition, adverse symptoms were attributable to treatment in an average of up to 10% of individuals, depending on the drug class, for those receiving antihypertensives at standard dose, according to a meta-analysis of randomized trials.^2^ Our model’s success rates, which include the likelihood of blood pressure control and failure because of adverse effects fall in these ranges.

The advantage of combination therapy in successfully achieving blood pressure control without adverse effects is supported by a meta-analysis of randomized trials, which found that the blood pressure lowering effects of different drug classes are additive, whereas adverse symptom effects are less than additive.^2^ We found that for a diversity of individuals for whom our model predicts higher success with combination therapy, the recommendation holds regardless of pretreatment blood pressure levels. Combination therapy is typically started for more severe cases, which would potentially bias our model against combinations as the treatment likelihood is smaller. However, our results show that ACE inhibitor-thiazide combination is still the preferred treatment in a wide variety of cases.

Limitations of our algorithm include small population size of Black and other non-White individuals in the training and validation sets. Studies have shown worse blood pressure response with ACE inhibitors in Black patients as compared with White patients,^14^ which support our findings. In the general Black population, the recommendation is to start initial antihypertensive treatment with a thiazide-type diuretic or CCB.^10^ Despite the small proportion of Black individuals in our model training population our results support this recommendation.

Our model results do not directly account for long-term patient outcomes as success is defined by 1 year blood pressure control. This limitation is mitigated as the physicians account for long-term outcomes when deciding on primary treatment in our data set and our model results are highly aligned with the clinical guidelines. Guidelines and particularly blood pressure targets change with the availability of new evidence. It is possible that the current model results, which are optimized to JNC 8 blood pressure targets may not be fully applicable if treatment success is defined by other targets. In addition, as we developed the model within a retrospective causal inference framework, it is necessary to further validate our results with a randomized controlled clinical trial.

## Conclusion

In conclusion, in this study we developed and validated a causal, DNN-based machine learning algorithm that personalizes hypertension management. Our findings suggest that our model can be leveraged to increase rates of blood pressure control in the population.

## Potential Competing Interests

Given his role as Editor-in-Chief, Dr Francisco Lopez-Jimenez, had no involvement in the peer-review of this article and had no access to information regarding its peer-review. Full responsibility for the editorial process for this article was delegated to an unaffiliated Editor.
