# Supplementary material for: Causal Deep Neural Network-Based Model for First-Line Hypertension Management
Source: Mayo Clin Proc Digit Health. 2023 Nov 30;1(4):632–40. doi: 10.1016/j.mcpdig.2023.10.001 (PMC11975727; doi:10.1016/j.mcpdig.2023.10.001)
Supplement: Supplementary Tables and Figures [file mmc1.pdf]

Supplementary Materials

Supplementary Table 1. ICD-10 Diagnosis Code Validity on Training Set Using Representative Conditions Defined by Objective Metrics
Supplementary Table 2. Hypertension Causal, Deep Neural Network Model Architecture
Supplementary Table 3. Most Likely and Most Successful Drug Classes for White and Black Patients by Comorbidity in the Training Set
Supplementary Table 4. Performance of Different Models on Validation Set
Supplementary Figure 1. Model Confidence as a Function of Performance
Supplementary Figure 2. Treatment Success Likelihood for ACE Inhibitor vs Thiazide as Monotherapy or Combination Therapy as a Function of Creatinine
Supplementary Figure 3. Treatment Success Likelihood for ACE Inhibitor vs Calcium Channel Blocker vs Thiazide as a Function of Age for Black Individuals

Supplementary Table 1. ICD-10 Diagnosis Code Validity on Training Set Using Representative Conditions Defined by Objective Metrics

**ICD-10 code linked condition**	**Metric to validate ICD-10 comorbidity**	**% Training set cases with positive metric**	**Calculation details**
Hypertension	≥130 systolic or ≥80 diastolic[1]	66.8	Average of ≥ 2 blood pressure readings within 1 year prior to hypertension diagnosis
Type II Diabetes Mellitus	HbA1c ≥6.5%	66.4	Value included any time prior to hypertension diagnosis to 1 year post-diagnosis
Chronic Kidney Disease	GFR <60 mL/min/1.73m2	87.4	2009 CKD-EPI equation used to calculate GFR.[2] Value included any time prior to hypertension diagnosis to 1 year post-diagnosis

Supplementary Table 2. Hypertension Causal, Deep Neural Network Model Architecture

**Model**	**Architecture**
Treatment likelihood model	batch norm<br>linear layer (21 neurons)<br>sigmoid<br>batch norm<br>linear 2 (63 neurons)<br>sigmoid<br>linear 3 (780 neurons)
Success model	batch norm<br>linear 1 (63 neurons)<br>sigmoid<br>batch norm<br>linear 2 (315 neurons)<br>sigmoid<br>linear 3 (1 neuron)

For the success probability calculation 20 such models were used. Each model was initiated with different weights and was trained on 80% of the train set. The median score of the 20 models defined the score and (un)confidence was defined as ([90 per] - [10 per]) / median. Unconfidence scores above 0.55 were labeled as "low confidence", scores between 0.55 and 0.40 were labeled as "medium confidence", and scores below 0.40 were labeled as "high confidence".

Supplementary Table 3. Most Likely and Most Successful Drug Classes for White and Black Patients by Comorbidity in the Training Set

Comorbidity	Most common treatment - white (%)	Most successful treatment - white (average % success)	Most common treatment - black (%)	Most successful treatment - black (average % success)
All patients	ACEi (26.7)	ACEi/TZ (45.3)	CCB (21.6)	TZ (33.3)
Tachyarrhythmia	BB (22.8)	ACEi/TZ (42.3)	Low numbers	Low numbers
Type II diabetes	ACEi (34.2)	ARB/TZ (42.7)	Low numbers	Low numbers
Ischemic heart disease	BB (27.0)	TZ (40.8)	Low numbers	Low numbers
Asthma	ACEi (25.4)	ACEi/TZ (41.4)	Low numbers	Low numbers
Chronic kidney disease	ACEi (16.9)	ACEi (37.1)	Low numbers	Low numbers
Congestive heart failure	BB (17.9)	ACEi (16.3)	Low numbers	Low numbers
Gout	ACEi (27.4)	ACEi (38.6)	Low numbers	Low numbers
None	ACEi (29.4)	ACEi/TZ (47.0)	CCB (26.4)	TZ (34.2)

Success results shown if the patient counts were >35 and >2% of total cases in the category. ACEi = angiotensin converting enzyme inhibitor. ARB = angiotensin receptor blocker. BB = beta blocker. CCB = calcium channel blocker. TZ = thiazide.

Supplementary Table 4. Performance of Different Models on Validation Set

Model	Precision	Recall	F1
Statistical model	51.6	19.3	28.1
XGBoost	46.4	37.4	41.4
Hypertension causal, deep neural network model	51.7	44.4	47.8

Supplementary Figure 1. Model Confidence as a Function of Performance

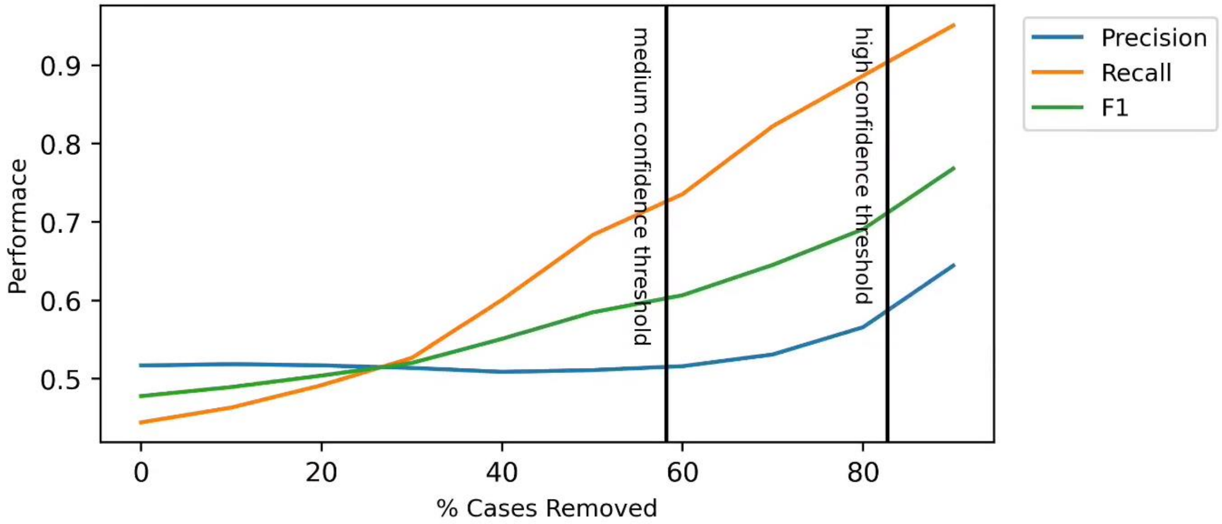

The validation cases were sorted by model confidence. Cases were removed in the order of lowest to highest confidence value. The x-axis is the percentage of cases removed. The vertical lines indicate our definition of medium and high model confidence. All cases to the right of these lines indicate cases that are in that confidence level or higher.

Supplementary Figure 2. Treatment Success Likelihood for ACE Inhibitor vs Thiazide as Monotherapy or Combination Therapy as a Function of Creatinine

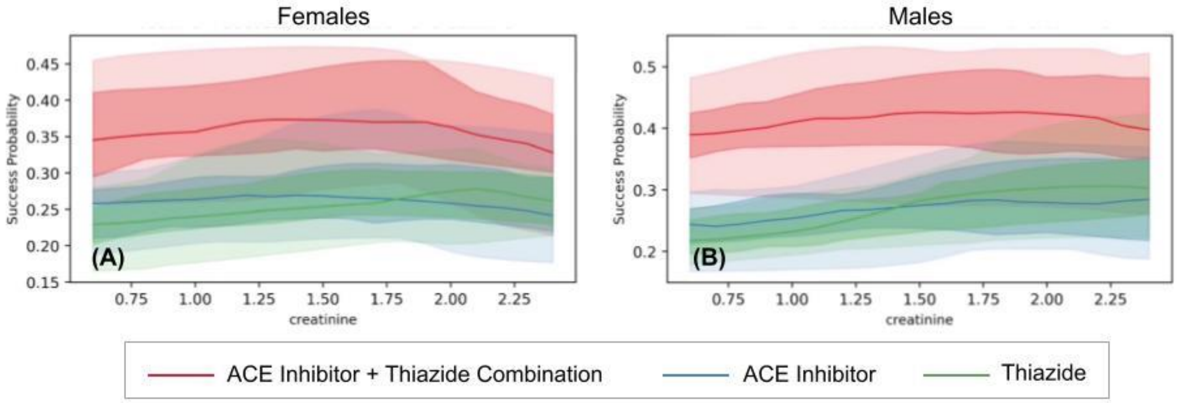

Model prediction of success rates as a function of a creatinine by treatment drug class (top three most successful per feature set). Representative cases shown in graphs are for a white 50 year-old with no comorbidities and pretreatment blood pressure of 160/90. The success prediction is composed of 20 models. Each was trained on 80% of the train set and was initiated with different weights. The median score of these models was considered to be the actual probability and is the dark line in the plots. The medium tones represent the area between the 25th and the 75th percentiles and the lighter tones represent the area between the 10th and 90th percentiles.

Supplementary Figure 3. Treatment Success Likelihood for ACE Inhibitor vs Calcium Channel Blocker vs Thiazide as a Function of Age for Black Individuals

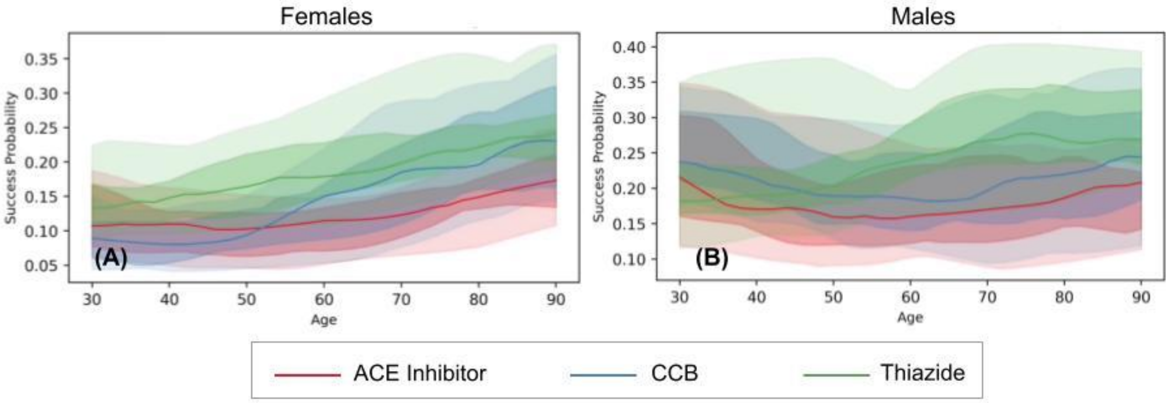

Model prediction of success rates as a function of age by treatment drug class (top three most probable treatments per feature set). Representative cases shown in graphs are for black individuals with no comorbidities and a creatinine of 0.8 mg/dL. The success prediction is composed of 20 models. Each was trained on 80% of the train set and was initiated with different weights. The median score of these models was considered to be the actual probability and is the dark line in the plots. The medium tones represent the area between the 25th and the 75th percentiles and the lighter tones represent the area between the 10th and 90th percentiles.

References

1. Whelton PK, Carey RM, Aronow WS, et al. 2017 ACC/AHA/AAPA/ABC/ACPM/AGS/APhA/ASH/ASPC/NMA/PCNA Guideline for the Prevention, Detection, Evaluation, and Management of High Blood Pressure in Adults: A Report of the American College of Cardiology/American Heart Association Task Force on Clinical Practice Guidelines. *J Am Coll Cardiol.* 2018;71(19):e127-e248.
2. Levey AS, Stevens LA, Schmid CH, et al. A new equation to estimate glomerular filtration rate. *Ann Intern Med.* 2009;150(9):604-12.
